# OPTIMIZING POLYPHARMACY CARE: INSIGHTS FROM PHARMACISTS INFORMING A POPULATION HEALTH APPROACH

**DOI:** 10.1093/geroni/igae098.2842

**Published:** 2024-12-31

**Authors:** Nicha Tantipinichwong, Dina Mustafa, Irina Avidon, Sonja Rosen, Michelle Keller

**Affiliations:** Cedars-Sinai Medical Network, Los Angeles, California, United States; Cedars-Sinai Medical Care Foundation, Los Angeles, California, United States; Cedars-Sinai Medical Care Foundation, Los Angeles, California, United States; Cedars-Sinai Medical Center, Los Angeles, California, United States; University of Southern California, Los Angeles, California, United States

## Abstract

Over 60% of older adults grapple with multimorbidity (more than one chronic condition), while polypharmacy (taking four or more medications) is prevalent in 65% of cases. Polypharmacy correlates with adverse outcomes such as mortality, falls, fractures, hospitalizations, and decline in functionality and cognition. Many health systems lack systematic monitoring of polypharmacy and its consequences. To address this, we aimed to develop a population health outcomes dashboard for a large urban health system, focusing on polypharmacy outcomes. We also aimed to increase the effectiveness of our polypharmacy consults. Our qualitative study, employing Framework Analysis, involved interviews with eight clinical pharmacists conducting polypharmacy medication review consults for older adults. We aimed to identify challenges, enhance clinic efficiency, and pinpoint data and outcomes for tracking on the dashboard. Major challenges included time constraints due to lengthy medication lists, lack of documentation of herbal supplements on medication lists, and patients’ readiness for consultations. Opportunities for efficiency improvement included pre-consultation patient preparation, streamlined note templates, and prioritizing intervention elements in each visit. Beyond clinical and disease-related outcomes, valuable dashboard measurements encompassed patients’ and physicians’ perceived benefits (e.g., improved medication adherence). These findings will guide enhancements to the polypharmacy clinic workflow and provide insight for the development of the polypharmacy outcomes and interventions dashboard.

